# The female orgasm and the homology concept in evolutionary biology

**DOI:** 10.1002/jmor.21544

**Published:** 2022-12-15

**Authors:** Silvia Basanta, Laura Nuño de la Rosa

**Affiliations:** ^1^ Department of Evolutionary Biology University of Vienna Vienna Austria; ^2^ Department of Logic and Theoretical Philosophy Complutense University of Madrid Madrid Spain

**Keywords:** clitoris, evo‐devo, female orgasm, history of sexuality, homology

## Abstract

The definition of homology and its application to reproductive structures, external genitalia, and the physiology of sexual pleasure has a tortuous history. While nowadays there is a consensus on the developmental homology of genital and reproductive systems, there is no agreement on the physiological translation, or the evolutionary origination and roles, of these structural correspondences and their divergent histories. This paper analyzes the impact of evolutionary perspectives on the homology concept as applied to the female orgasm, and their consequences for the biological and social understanding of female sexuality and reproduction. After a survey of the history of pre‐evolutionary biomedical views on sexual difference and sexual pleasure, we examine how the concept of sexual homology was shaped in the new phylogenetic framework of the late 19th century. We then analyse the debates on the anatomical locus of female pleasure at the crossroads of theories of sexual evolution and new scientific discourses in psychoanalysis and sex studies. Moving back to evolutionary biology, we explore the consequences of neglecting homology in adaptive explanations of the female orgasm. The last two sections investigate the role played by different articulations of the homology concept in evolutionary developmental explanations of the origin and evolution of the female orgasm. These include the role of sexual, developmental homology in the byproduct hypothesis, and a more recent hypothesis where a phylogenetic, physiological concept of homology is used to account for the origination of the female orgasm. We conclude with a brief discussion on the social implications for the understanding of female pleasure derived from these different homology frameworks.

## Graphical abstract

The homology concept and its application to reproductive/genital structures, and the physiology of sexual pleasure, has a tortuous history. While there is a current consensus on the developmental homology of genital and reproductive characters, no agreement exists on the physiological translation, neither on the evolutionary origination and roles, of these correspondences and their divergent histories. We analyze the impact of evolutionary perspectives on homology as applied to the female orgasm, and their consequences for biological and social representations of female sexuality.

**Image Reference**: Bonamy, C., P. Broca, and E. Beau. *Atlas d'anatomie descriptive du corps humain*, Vol. III, Paris, Masson et Cie., 1850.

Public domain

*Source*: https://www.digitale-sammlungen.de/en/view/bsb10330713?page=257

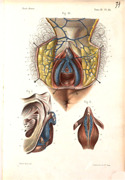

## 1 INTRODUCTION

Scientific controversies on the female orgasm condense how gender biases have shaped research on female anatomy and physiology, and how biological sciences, in turn, have both constrained and enabled new narratives on human sexuality. Far from being a resolved topic, the biomedical representation and explanation of the female orgasm is a matter of lively debate in various biological disciplines concerned with the study of sexuality, including medical anatomy, animal endocrinology and neurophysiology, and evolutionary biology. Previous studies by gender and sexuality scholars have documented various episodes of the history of ideas about female genitalia and the physiology of pleasure (e.g., Laqueur, 1986; Lloyd, 2005; Moore, 2018, 2021; Tuana, 2004). This paper aims to complement these works by looking at how changing notions of homology have been used in evolutionary studies of female pleasure in the context of different societal assumptions and expectations on female sexuality.

Textbook depictions of male and female urogenital systems in mammals agree in presenting them as biological homologs (e.g., Carlson, 2018, pp. 394–401). In the early stages, mammalian embryos have indifferent structures that develop into male or female derivatives. From an undifferentiated sexual duct system, it is the paramesonephric (Müllerian) ducts that further develop in the case of a female embryo. In males, it is the mesonephric (Wolffian) ducts that give rise to the structures encompassing the epididymis, seminal vesicles, and ejaculatory duct. The genital ridge develops into ovaries or testes, the primordial germ cells into ova or spermatozoa, and the sex cords into granulosa or Sertoli cells. The external genitalia also develop from an undifferentiated condition: the genital tubercle develops into the glans penis or the clitoris, the urogenital fold becomes the spongy urethra in males and the labia minora in females, and the genital swellings give rise to the scrotum in males and the labia majora in females.

The definition of homology and its application to reproductive structures, external genitalia, and the physiology of sexual pleasure has a tortuous history, and while nowadays there is a wide consensus on the developmental homology of genital and reproductive structures, there is no agreement on the physiological translation, neither on the evolutionary origination and roles, of these structural homologies. This article analyzes the impact of the comparative perspective in biomedical representations of female sexuality and female reproduction since the 19th century to contemporary debates on the role and nature of the female orgasm. More specifically, we dig into evolutionary perspectives of homology and their consequences for the biological and social understanding of female sexuality and reproduction.

The structure of the article will be as follows. In Section 2, we survey the history of pre‐evolutionary biomedical views on sexual difference and sexual pleasure. Section 3 refers to the shaping of the concept of sexual homology by comparative anatomists in a new phylogenetic framework towards the end of the 19th century. Section 4 analyses debates on the anatomical locus of female pleasure at the crossroads of theories of sexual evolution and new scientific discourses in psychoanalysis and sex studies. Section 5 moves back to evolutionary biology and examines the consequences of neglecting homology in adaptive explanations of the female orgasm. The last two sections investigate the core role played by different articulations of the homology concept in evolutionary developmental explanations of the origin and evolution of the female orgasm. Section 6 looks at the role of sexual homology in articulating the byproduct hypothesis. Section 7 examines a more recent hypothesis where a phylogenetic, physiological concept of homology, is applied to account for the origination of the female orgasm. We conclude with a brief discussion on the societal implications for understanding female pleasure derived from these different homology frameworks.

## 2 EROTIC HOMOLOGY AND REPRODUCTIVE HOMOLOGY IN PRE‐EVOLUTIONARY TIMES

Before the rise of comparative anatomy in the 19th century, the recognition of identity relationships between individual organisms relied either on analogical reasoning, or on intuitive understandings of “sameness,” rather than on any formal criteria of homology. Nonetheless, since Aristotle, comparative studies used different informal criteria based on the number, relative size, and position of organs, to characterize the “unity in diversity” of animal form. Identity relationships included those holding between the sexes, what we refer today as sexual homology, namely an instance of serial homology that captures the relation of identity between traits belonging to the two sexes of the same species (Fusco, 2022). Different conceptualizations of sexual homology constituted the conceptual framework where biomedical representations of human sexuality were forged before the advent of evolutionary thought. According to an influential historiographical narrative founded by Thomas Laqueur (1986, 1992), homology thinking dominated biomedical views on human bodies from Greco‐Roman antiquity to the late 17th century. Under the “one‐sex model,” male and female bodies were regarded as instantiations of the same type, while differing in degree of development, topological position, and relative value. In contrast, the economic, political, and cultural transformations of the 18th century led to a new articulation of the differences between the sexes. In the “two‐sexes model,” bodies started to be understood as essentially sexed, and sexual traits came to be clustered into two distinct natural kinds. Although this dichotomization affected all aspects of human anatomy, genitalia became the primary foundation of sexual dimorphism.

In the last few decades, historians of gender and sexuality have objected that Laqueur's two‐stages narrative is built upon scarce and biased sources, and simplifies a much more nonlinear and complex history in which the themes of sameness and difference between the sexes cohabited in different time periods (Harvey, 2002; King, 2013; Linton, 2022; Stolberg, 2003).^1^ Importantly for the case of the female orgasm, Alison Moore (2018) has convincingly shown that two different versions of the homologous model cohabited well into the 19th century. In the Galenic tradition, which Laqueur takes as a reference framework, female genitalia were regarded as inverted versions of male genitalia, the testes being homologous to the ovaries and the penis to the vagina. However, the Hippocratic tradition considered the clitoris, not the vagina, as homologous to the penis. While both accounts regarded genital structures as anatomically homologous in the two sexes, their implications for the conceptualization of female pleasure were radically distinct: “in the Galenic view, women's pleasure is minimized and assumed to follow directly from coitus, while in the Hippocratic view women's pleasure is emphasized and located outside the zone of direct coital reception” (Moore, 2018, p. 55). As a consequence, the Hippocratic model permitted comparing sexual pleasure in men and women independently of their differing reproductive structures. In contemporary terms, the clitoris‐penis homology allowed to separate *reproductive* homology from *erotic* homology.

Nonetheless, it is generally agreed that the Galenic and the Hippocratic traditions shared the belief that orgasm, or at least sexual arousal, had an analogous reproductive role in the two sexes. Generative substances were thought to be produced as a result of intercourse, and cause some kind of pleasant sensation when released in both men and women. The mammalian egg was only identified by Karl Ernst von Baer in 1827, and less than two decades later, the discovery of spontaneous ovulation led to the recognition of a radical separation between sexual pleasure and reproduction in women, as opposed to men (Laqueur, 1986). Up to then, the accumulated observations on induced ovulation and the use of the rabbit as a model organism in reproductive biology had only confirmed the assumption on a close link between pleasure and generation in females. In 1843, Theodor L. W. Bischoff reported the first unfertilized mammalian egg and found scars from ovulation in a dissected female dog that had not experienced any coitus. Bischoff's discovery was the first widely recognized evidence for spontaneous ovulation, and led to the conclusion that intercourse and ovulation were not necessarily linked. According to Laqueur, the discovery of spontaneous ovulation boosted the crisis of the homologous model as the primary way of representing the female body (1992, p. 213). Further research in reproductive biology, particularly in the cytological depictions of sperm and egg cells in mammals, might have contributed to what came to be perceived as fundamental differences between the anatomy and physiology of males and females. Thus, new embryological depictions of sperm and egg as active and passive, respectively, also influenced how new scientific narratives portrayed male and female pleasure as substantially different (McLaren, 2002, p. 337). Importantly, this new paradigm ceased momentarily with the search for a direct physiological mechanism connecting recreational and procreational sex in the female body. Nonetheless, the interest of physicians in women's pleasure did not disappear. Quite the contrary, medical writing on women's pleasure continued to be prevalent in the French context during the second half of the 19th century. Motivated by the belief that women had an equal potential for sexual pleasure, doctors continued to worry about women not having orgasms in marriage because they thought it would result in divorce and contribute to declining birth rates (see Cryle & Moore, 2011).

## 3 FROM SEXUAL HOMOLOGY TO PHYLOGENETIC HOMOLOGY

With the rise of comparative anatomy and embryology in the 19th century, a new concept of homology strongly burst in the conceptualisation of sexual difference. In his celebrated conceptual taxonomy, Richard Owen (1843) distinguished between three kinds of correspondences of body parts, namely that between species (special homology), that between repeated elements within the body of an individual (serial homology), and that between a character of a species with that of the archetype (general homology). All these three homology relations had an influence on how sexual differences were represented and explained in pre‐evolutionary comparative anatomy. Firstly, the correspondences between the sexes came to be conceptualized as an instance of homology, and more specifically as a subtype of serial homology. Secondly, sexual differences were homologized across species. Finally, sexual differences were conceptualized as resulting from the differentiation of a bisexual archetype represented by hermaphrodism. In the 1830s, the teratologist Isidore Geoffroy Saint‐Hilaire defined hermaphrodism as “the coexistence in the same individual of both sexes or of some of their characteristics” (1836, p. 31). Geoffroy Saint‐Hilaire relied on embryology to argue that the reproductive systems of males and females developed from a hermaphrodite stage, and that later in development, organs of one sex developed while those of the other sex remained rudimentary. The various types of hermaphrodism resulted from excess or defect of growth in the typical development of the reproductive organs of each sex (see Linton, 2022). The case of sexual differentiation instantiated a more general principle, namely the principle of compensation or the balancement law, according to which organs are enlarged at the expense of other organs that are rendered rudimentary (Appel, 1987, p. 76). Therefore, in the 19th century, comparative embryology allowed to understand sexual sameness from a comparative genealogical perspective. Sexual organs were homologous insofar as they “developed in a similar manner from a similar fundamental structure” (Watson, 1879, p. 52). The embryological definition of homology not only allowed the grounding of the identity relationships between external genitalia, but also between internal sexual organs. These included the testis and ovaries as derived from the genital ridge, the spermatozoa and ova from the primordial germ cells, the Sertoli and granulosa cells from the sex cords, and the male and female derivatives of the mesonephric tubules, ducts and ligaments, and the paramesonephric ducts. In the context of the theory of parallelism, remnants of sexual organs, such as incomplete uterus or vaginas in males, were identified in “lower animals”, as well as in human pathologies (Watson, 1879).

After the publication of the *Origins of species*, the transformation of homology into a historical or phylogenetic concept led to interpreting the previously established anatomical and embryological correspondences between the sexes as an instance of common ancestry. In the reedition of *Elements of comparative anatomy*, Carl Gegenbaur reinterpreted hermaphrodism as the ancestral stage preceding the evolution of sexual differentiation due to relative changes in the size of sexual organs. According to Gegenbaur, sexual differentiation not only affected generative substances and reproductive organs, but entailed an integral differentiation of individuals: “The separation of the sexes affects the whole of the organism, for it produces a series of changes in each sex, which affect organs that had primitively little to do with the sexual function. Sexual differentiation is completed when the two kinds of organs are given over to different individuals. Thenceforward for reproduction, not only two different substances, semen and ova, and two different organs for producing them, are necessary, but also two individuals; these are distinguished as male and female” (Gegenbaur, 1878, p. 54).

In *The Descent of Man*, Darwin credited Gegenbaur's hypothesis of the hermaphrodite ancestor of vertebrates, although he also speculated that rudimentary sexual traits in mammals might have resulted from hereditary correlations between the sexes: “to account for male mammals possessing rudiments of the accessory female organs, and for female mammals possessing rudiments of the masculine organs, we need not suppose that their early progenitors were still androgynous after they had assumed their chief mammalian characters. It is quite possible that as the one sex gradually acquired the accessory organs proper to it, some of the successive steps or modifications were transmitted to the opposite sex” (Darwin, 1871, p. 208). According to Ghiselin, these early speculations on the hermaphrodite ancestor reflect a conflation between sexual and phylogenetic homology, and Darwin's hesitation between phylogenetic and developmental explanations of sexual homology illustrate the incipient historical decoupling between these two homology concepts (Ghiselin, 1969, pp. 118–119, and Ghiselin, 2005).^2^

The concept of embryological homology also joined the discussion on the evolutionary origins of sexual differentiation. Haeckel's biogenetic law was the core conceptual framework to connect the development and evolution of sexuality. In this new context, embryonal sexual differentiation was believed to reflect the phylogenetic history of mammals, and some sexual characters were interpreted as vestigial traces of the evolutionary past. “Atrophied” sexual organs, that is, organs reduced in size as compared to their homologous counterparts, such as male breasts as compared to females, or glans clitorises as compared to penis, were reinterpreted as vestigial characters. On many occasions, the development and evolution of sexual characters were regarded as progressive, teleological processes. Evolutionary teleological explanations of human sexuality were embedded in the wider historical teleological theory of sexuality typical of the 19th century, first formulated in humanistic fields outside evolutionary biology (Moore, 2021).^3^ The early 20th‐century debates on the anatomical locus of female pleasure in the field of sexology were highly influenced by this historical interpretation of the *telos* of human sexuality. These included, among others, Havelock Ellis' ideas on bisexuality, Gregorio Marañón's reflections on gender, and Magnus Hirschfeld's concept of primeval inter‐sexuality (Bauer, 2012; Moore, 2015). In the following section, we concentrate on the interaction between psychoanalysis and evolutionary perspectives on human sex. We will contrast this approach with the later quantitative studies of human sexuality, which were influenced by a different, quantitative school in evolutionary biology, and set up the context for contemporary evolutionary debates on the female orgasm.

## 4 SEXUAL HOMOLOGY AND THE MOVING LOCUS OF FEMALE PLEASURE

While anatomical homologies between genitalia were well‐established, differing views about the anatomical locus of female sexual pleasure continued circulating throughout the 19th century, and it was not until the early 20th that the questioning of clitoral orgasm became mainstream in medical practice. Socio‐political imperatives on the distinction between men and women, together with new biomedical views of sexuality were involved in this transition (Moore, 2018). These included dismissive medical views of masturbation, but the psychoanalytic theory of the vaginal orgasm was undoubtedly the most influential scientific theory in this regard. In his *Three essays on the theory of sexuality*, Freud (1905/2017) argued that, while men remain consistent in retaining their penis as the core anatomical locus of sexual pleasure, women experience in their transition to maturity a “transfer” of their center of sexual sensitivity from the clitoris to the vagina.

Freud's transfer theory reconciled two modern views on sex, namely the theory of bisexuality, and the distinction between males and females based on their complementary roles in reproduction (Freud, 1905/2017, p. 142). At various places in his works, Freud notes that sciences do not provide a clear‐cut distinction between the sexes, and interprets this anatomical fuzziness as an indication of a shared, primary bisexuality: “(science) draws your attention to the fact that portions of the male sexual apparatus also appear in women's bodies, though in an atrophied state, and vice versa in the alternative case. It regards their occurrence as indications of bisexuality, as though an individual is not a man or a woman but always both‐merely a certain amount more the one than the other” (Freud, 1964, p. 114).

The themes of bisexuality, hermaphrodism, and androgyny were ubiquitous within and outside the scientific circles of Fin‐de‐siècle Vienna (McEwen, 2012). In his training as a biologist, Freud became influenced by the theory of bisexuality from multiple perspectives that deeply influenced his conception of human sexuality (Freud, 1905/2017, p. 142). These included the work of the physiologist Wilhelm Fliess on the “bisexual constitution” of every living organism (Heller, 1981), the comparative studies on hermaphroditism by the zoologist Carl Claus (Sulloway, 1992), and Darwin's theory on the original bisexuality of humans (Bauer, 2012). Under the Darwinian perspective, bisexuality (understood as the coexistence of male and female traits in the same individual) corresponded to an earlier, undifferentiated stage of development and evolution, while the progressive differentiation between the sexes was the result of natural selection (see Angelides, 2001; ch. 2).

The influence of historical explanations of sexuality can be found in Freud's reliance on teleological notions on how underlying homologies gradually diverge throughout evolution (Moore, 2021): genital homologies “lead us to suppose that an original bisexual disposition has, in the course of evolution, become modified into a unisexual one, leaving behind only a few traces of the sex that has become atrophied” (Freud, 1905/2017, pp. 243–244). A later evolutionary legitimation of the transfer theory with an important impact on psychoanalysis was proposed by the ethologist Frank A. Beach in the 1940s. According to this theory, the vaginal orgasm was a recent evolutionary acquisition of the human species, linked to the evolution of higher intellectual abilities related to the self‐control of sexuality (Beach, 1948). This explained that only a few women were able to experience orgasm, and transformed the vaginal orgasm into an “evolutionary ideal” (Sherfey, 1966).

Freud's transfer theory departed from the widely held recognition of embryological, anatomical, and phylogenetic homologies between the genitals of the two sexes, but required the fragmentation of female pleasure into two distinct erogenic zones. While the clitoris was seen as a male part in women's bodies, the vagina constituted a distinctive female part without a male homologous correlate. This anatomical differentiation within the female genital system had no precedent in previous theories on the anatomical basis of female pleasure (Tuana, 2004). Physiological homology, in Freud's eyes, was linked to psychological homology, insofar as each genital zone was associated to the distinct sexual behaviors attributed to the two genders (Traub, 2001). Therefore, the transition of libido from the clitoris to the vagina permitted to overcome anatomical homology and achieve a psycho‐physiological differentiation. As many commentators have noted, this postulated transition was not a descriptive, but a normative one “attempting to reconcile women's physiology with a heterosexual imperative” (Traub, 2001, p. 153). At the turn of the 20th century, sexuality constituted in Vienna the primary idiom through which topics and anxieties related to modern society were reflected (Luft, 2003), and Freud's theory can only be understood within this cultural context. Thus, according to the transfer theory, the proper form of femininity was achieved by restraining sex to its reproductive function, and the lack of vaginal orgasms during intercourse was theorized as the failure of culture to resignify women's bodies into their appropriate societal roles (see Koedt, 1970; Laqueur, 1986; Moore, 2018).

The Freudian transfer theory constituted the basis for dominant narratives of female sexuality in the following decades, portraying women as either lacking sexual passions or as victims of pathological affections (Maines, 2001). After the influence of Freudian ideas in the post‐war, North American context, frigidity was redefined and diagnosed as a lack of orgasm in penetration (Cryle & Moore, 2011). In such a context, a new generation of sexologists stood up for the modern expectation of an egalitarian depiction of sexual pleasure, and resorted to the concept of sexual homology as a reaction against the psychoanalytic theory (Gerhard, 2000; Moore, 2018). Nonetheless, Freud recognized his limitations as a man in theorizing on female pleasure and hoped his female disciples would elaborate further on the topic. The resulting tension is illustrated in the complex figure of Marie Bonaparte (1949). On the one hand, Bonaparte wanted to embrace the androgyny that psychoanalysts read on clitoral pleasure, interpreting it as a positive sign of the gender equality brought about by modern civilization. On the other hand, she assumed the Freudian dichotomization of erogenous zones in the female body, and applied its normative implications to her own. By surgically relocating her clitoris, she attempted to conciliate masculine and female pleasure (Cryle & Moore, 2011, pp. 222–247).

In the early 1950s, Alfred Kinsey and his group at the Institute for Sex Research distanced themselves from the psychoanalytic approach to sexuality, and embraced an empirical, statistical approach that prioritized quantitative data over subjective and individual case studies. Kinsey decided to study human sexual behavior after a failed career as an evolutionary taxonomist. Although this meant focusing on the proximate causes of sexual behavior, he kept in touch with debates precipitating into the Modern Synthesis, and applied the methods for data gathering and ordering, as well as the processual approaches learned from evolutionary biology, to human sex research (Drucker, 2014). In a groundbreaking treatise, Kinsey and collaborators defended the clitoral basis of the female orgasm by relying again on the embryological homology between male and female genitalia: “In the female and male mammal the external reproductive organs, the genitalia, develop embryologically from a common pattern. They are, therefore, homologous structures in the technical meaning of the term” (Kinsey et al., 1953/1998, p. 571). In their view, the comparison of female and male sexual behavior depended on a “better understanding of the anatomy and physiology of sexual response and orgasm” (p. 575). After five chapters devoted to comparing the anatomy and physiology of human sexuality in both sexes, they concluded that there was no clear way to classify men and women into two different sexual groups in terms of orgasmic capacity, sexual behavior, or body composition: there was one single orgasm in females, and it was homologous to the male orgasm.

A decade later, William Masters and Virginia Johnson continued this turn toward an empirical approach to human sexuality, publishing the results of their physiological experiments in their 1966 book *Human Sexual Response*. Reporting on Kinsey and colleagues' research, Masters and Johnson argued for the role of the clitoris in the female orgasm on the basis of anatomical and physiological homologies between male and female sexual response. Masters and Johnson considered the psychological and physiological dimensions as integrated aspects of the female orgasm: “For the human female, orgasm is a psychophysiologic experience occurring within, and made meaningful by, a context of psychosocial influence. Physiologically, it is a brief episode of physical release from the vasocongestive and myotonic increment developed in response to sexual stimuli. Psychologically, it is a subjective perception of a peak of physical reaction to sexual stimuli” (Masters and Johnson, 1966, p. 127). However, they developed methods to describe and measure the physiological and psychosocial dimensions of the human orgasm independently. Along with the subjective communication of sensory experience, ways of measuring the physiological processes described were needed for turning orgasm into an objective and comparable trait. Their major contribution in this regard was the proposal of a “human sexual response cycle” comprising four phases of sexual response—excitement, plateau, orgasm and resolution. Assuming that the sequence of physiological changes was the same in both sexes, Masters and Johnson identified and measured those neurological, muscular, and vascular parameters that were comparable in men and women, concluding that male and female orgasms were homologous in terms of duration, intensity, and underlying mechanisms.

In concluding that the clitoris was the main erogenic zone in the female body, human sex research from the 1950s and 1960s had a great impact on depathologizing female pleasure in biomedical studies and treatments of human sexuality. In addition, between the late 1960s and the mid‐1970s, there was an explosion of feminist analyses of the political meaning of sexuality that critiziced the heteronormative biases of psychoanalysis and relied on the new “facts of biology” to reclaim women's sexual autonomy (Gerhard, 2000).^4^

From a comparative scope, sex studies of the female orgasm had a revolutionary impact as well. The new sexologists tended to assume that the female orgasm was specific to humans and therefore did not discuss it in an evolutionary context. Nonetheless, their focus on anatomy and function, and the description of orgasm as a sequence of physiological events, set the basis for objective definitions of sexual response, allowing for the study of the female orgasm as a natural trait that could be found outside the human species (Musser, 2012). As a matter of fact, although Kinsey and colleagues concluded that “orgasm is infrequent and possibly absent among females of most species of mammals” (Kinsey et al., 1953/1998, p. 135), they did refer to endocrinology reports of female orgasms in other species,^5^ and admitted that the main obstacle for testing its existence was a lack of adequate criteria for its identification and interpretation outside humans (pp. 628–629).

Between the late 1960s and early 1970s, primatologists and anthropologists such as Suzanne Chevalier‐Skolnikoff started to study behavioral signs indicating orgasmic activity in female primates (see Musser, 2012). In turn, comparative studies of sexual response prepared the terrain for an evolutionary approach to the female orgasm. On the one hand, studies of sexual behavior in primates opened the door for evolutionary interrogations on the adaptive role of the female orgasm. On the other hand, the critics of adaptationism started challenging selectionist explanations of the female orgasm, bringing homology back to evolutionary explanations of female sexuality.

## 5 THE FEMALE ORGASM AS AN EVOLUTIONARY ADAPTATION

In the early 20th century, the fall of evolutionary morphology and the acceptance of natural selection as the only guiding force of evolution, led to a proliferation of adaptive approaches to human sexuality that pushed homology thinking to the background. In the Darwinian view of evolution, homologous parts differed through adaptation, not only with the external and internal environment, but also with parts of the other sex. In this new explanatory context, the theory of sexual selection became the core evolutionary force accounting for sexual differentiation and had major implications for the representation of female sexuality. Placing female preferences and choice at the explanatory center challenged the passionless Victorian depiction of female sexuality (Thornhill & Gangestad, 2008, p. 3), but also reinforced the myth of the monogamous, “coy” female courted by undiscriminating males. Female promiscuity only started to be considered in the late 1970s, when the female perspective was introduced in evolutionary discussions on human sexuality (Hrdy, 1981/2009). For instance, genetic hypotheses for polyandry alleged that the extended sexual receptivity of female primates, as provided by their clitoral ability to experience orgasms, was a mechanism selected to generate genetic variation through mating with multiple males. However, under the assumption that reproductive functions should explain the evolution of female sexuality, the female orgasm became an evolutionary mystery (Buss, 2016), or “an adaptive paradox” (Kennedy & Pavličev, 2018). The physiological complexity of the female orgasm made the reasons for the evolutionary maintenance of this character even more mysterious, given that nonfunctional traits are expected to deteriorate unless they are under selection.

To solve this paradox, the earliest adaptive hypotheses attempted to unravel a direct role of the female orgasm in reproduction. In *The naked ape*, Desmond Morris speculated that the exhausting satisfaction following orgasm in women had the effect of keeping their bodies horizontal and retaining the seminal fluid (Morris, 1967, p. 79). Later in the 1990s, evolutionary biologists recovered a physiological hypothesis dating back to 1854 that postulated a link between female orgasm and sperm transport (see Levin, 2011a,b). After the discovery of spontaneous ovulation, the role of uterine contractions in assisting sperm transport replaced induced‐ovulation as the main mechanism for reuniting orgasm and conception in the female body. The new evolutionary versions of the upsuck hypothesis postulated that uterine contractions released by climax were functional in retaining the sperm inside the reproductive tract, thus promoting sperm competition (Baker & Bellis, 1993; Thornhill et al., 1995). The upsuck hypothesis was popularized by many authors in the 2000s (see Lloyd, 2005, pp. 216–217), but was widely discredited in the following decades. Despite some recent attempts to restore it (King et al., 2016), current evidence suggests that the female orgasm plays no role in sperm transport (Levin, 2011a), nor is there any correlation between female orgasms and offspring number (Zietsch & Santtila, 2011).

Alternative adaptive hypotheses have attempted to find an indirect role of orgasm in improving female reproductive success. Most rely on pair bonding, considering orgasm as an adaptation that motivates females to engage in intercourse outside the fertile phase of the cycle, and creates long‐term relationships with their male mates. First introduced by Morris (1967), different versions of the pair‐bonding hypothesis were formulated during the 1970s (see references in Lloyd, 2005, pp. 44–77). Nonetheless, the fact that vaginal intercourse alone is not the most reliable way to achieve orgasm in female primates, led advocates of pair‐bonding theories to emphasize mate selection (Alcock, 1978, 1980; Nebl & Gordon, 2022; Prum, 2017). Females would select mates arousing them to orgasm during intercourse, either because they just like it (Prum, 2017) or because they indirectly select for prosocial empathy (Kennedy & Pavličev, 2018).

In her influential book *The Case of the Female Orgasm*, Elisabeth Lloyd (2005) undergoes an exhaustive critical review of adaptive explanations of the female orgasm, outlining the theoretical and social biases shaping biological research. The core, general argument against adaptive hypotheses is their lack of solid evidence. Ultimately, selectionist theories on the female orgasm fail to meet standards of scientific corroboration, being an instance of untestable narrative explanations (Gould & Lewontin, 1979). The case of the female orgasm also illustrates how research paradigms lower the standards of good science whenever they meet the predictions of the theory they aim to corroborate, and preclude research in other directions. Thus, the statistical shortcomings of studies supporting the upsuck hypothesis passed widely unnoticed because they met the adaptationist expectation of a connection between female orgasm and reproduction, and adaptationist hypotheses were not even contrasted with alternative ones such as the byproduct hypothesis (see below). Secondly, Lloyd outlines the effects of the ancient, persistent social trend of considering female sexuality as inevitably tied to procreation. Adaptive theories look at the female orgasm during intercourse, and hence only regard it as a reproductive trait, rather than as just sexual behavior. Instead, Lloyd suggests considering sexuality as an independent set of activities “which are only *partially* explained in terms of reproductive functions” (Lloyd, 1993, 140). Examples of nonreproductive sexual behaviors include female same‐sex sexual behavior, or copulation outside the fertile phase of the ovarian cycle in mammals and birds (Thornhill & Gangestad, 2008).^6^

Evolutionary debates on the adaptive role of the female orgasm make scarce references to the anatomy, physiology, development, and phylogeny of female sexuality, the homology concept being virtually absent from the discussion. This style of reasoning reflects a general trend in adaptationist thinking. Insofar as the goal of selection‐based explanations is to understand why traits are preserved, the anatomy and physiology of sexual traits tend to be seen as irrelevant. The focus on selective pressures, together with the consideration of the female orgasm as a uniquely human trait, erased the comparative and phylogenetic dimensions of female sexual pleasure from evolutionary debates. Since the 1980s, the critique of adaptationism and the return of homology thinking has impacted evolutionary studies of human sexuality, and new hypotheses on the origin and evolution of the female orgasm have come to the fore.

## 6 BACK TO SEXUAL HOMOLOGY: THE BYPRODUCT HYPOTHESIS

In the late 1970s, the crisis of the adaptationist program led to a renaissance of the interest in homology in evolutionary biology that rapidly entered the debate over the female orgasm. Surprisingly enough, the first explanations of the female climax as a side‐effect of sexual homology (what later will be known as the byproduct hypothesis) can be found in the seminal works of the adaptationist approach to human sexuality. In *The naked ape*, Desmond Morris refers to the homology between clitoris and penis to speculate that the female orgasm might be, in origin, a “borrowed male pattern” (Morris, 1967, p. 80) that was later co‐opted for a different function. A decade later, Donald Symons advanced the idea of the female orgasm as a side‐effect of selection on male orgasm that was not in need of an independent adaptive explanation: “The female orgasm may be a byproduct of mammalian bisexual potential: orgasm may be possible for female mammals because it is adaptive for males” (Symons, 1979, p. 92). However, Symon's version of the byproduct hypothesis still entails an ambiguous concept of bisexuality, and seems to refer to a psychological or a behavioral concept of orgasm, rather than to any precise developmental, anatomical, or physiological notion of sexual homology (see Lee, 2013). The description of the female orgasm as the behavioral homolog of male ejaculation appears as well in several reports on macaques from the 1970s and 1980s (see references in West‐Eberhard, 2003; pp. 276–277). Nonetheless, the articulation of a developmental hypothesis on the evolution of the female orgasm seems to have required the reconceptualisation of homology in developmental terms that took place in the 1980s. In the frame of the nascent evolutionary developmental biology, or evo‐devo, two traits are homologous if they share a common developmental cause that explains their identity relation (Roth, 1984; Wagner, 1989). Relying on his previous criticisms of adaptationism and his advocacy for developmental and historical constraints, it was Stephen Jay Gould who provided the first evolutionary developmental version of the byproduct hypothesis. In invoking the female orgasm to advocate for nonadaptive explanations as legitimate explanatory alternatives, Gould (1987a) applied the classical notion of embryological homology to articulate an evolutionary explanation of the female orgasm. In Gould's view, the two sexes are “variants upon a single ground plan, elaborated in later embryology”. As a consequence, sexual differences do not need to be independently explained by adaptive criteria: just like “[m]ale mammals have nipples because females need them” (Gould, 1993, p. 83), females have a clitoris because males need their penis and the ejaculation associated to orgasm to reproduce. Ultimately, the evolutionary origin and maintenance of the female orgasm is a consequence of the selection on the male orgasm, and the associated constraints entailed by the common development of genitalia. In her 2005 book, Lloyd favored the byproduct hypothesis on the basis of the lack of evidence for adaptive explanations, and later contributed herself with new empirical evidence in support of the byproduct hypothesis. Together with Kim Wallen, Lloyd argued that higher variability in clitoral length from the shaft to the clitoral glans tip, as compared to the penis, suggests a lack of selective pressures (Wallen & Lloyd, 2008; but see Lynch, 2008).

The byproduct hypothesis is not free of problems, and constitutes a current matter of controversy. Objections can be grouped into two major categories, both related to the criteria and implications of relying on sexual homology to explain the evolution of the female orgasm. Firstly, genetic, anatomical, and physiological specificities of female sexual response cast doubts on the argument that the female orgasm can be exclusively explained as a byproduct of male physiology. These include the evolutionary preserved complexity and intensity of the female orgasm despite the lack of a function, the involvement of the pituitary in the female, but not in the male, orgasm (Huynh et al., 2013), and the lack of a clear sex‐genetic correlation for this trait, which suggests that different genetic factors underlie male and female orgasmic function and variance (Zietsch & Santtila, 2012; but see Wallen et al., 2012). Secondly, the existence of sexual homology does not preclude that of independent, selective pressures on the female orgasm. In her book *The Woman That Never Evolved*, Sarah Hrdy (1981/1999) claimed that Symons’ byproduct explanation dismissed female sexuality in this regard. Despite their anatomical homology, sexual organs have been subject to different selective pressures that are neglected by the critics of adaptationism: “we cannot explain special features of the clitoris such as its size, positioning, or degree of enervation merely by examining selection pressures on males to have a penis designed in a particular way. Once again, it was an error for evolutionists to assume glibly that by examining one sex we could learn all we needed to know about the other” (Hrdy, 1981/1999, p. 251). Gould's (1987) article also sparked a heated scientific exchange when John Alcock objected in a letter to the editor of *Natural History* (1978) that the clitoris and the female orgasm should not be conceptualized as a lesser, vestigial version of male anatomy and pleasure. In the Hrdy (1981/1999) bibliographical update of her book, Hrdy includes Gould's paper and criticizes him for the same reason: the byproduct hypothesis endorses and reinforces the assumption that female sexuality is a derived consequence of male sexuality, ultimately echoing the old view of sexuality being originally, and therefore fundamentally, masculine. In her reply to feminist critiques of the byproduct hypothesis, Lloyd warns about the perils of conflating biological function with social value: arguing for or against the hypothesis that the female orgasm is an adaptation does not say anything about the value of the trait (Lloyd, 2005, pp. 139–143).

Even if the notion of sexual homology grounding the byproduct hypothesis does not exclude a phylogenetic perspective, the reference to developmental correlations between the sexes does not allow itself to situate the female orgasm in a historical framework. In the last years, evo‐devo studies of female sexuality have gone beyond the concept of sexual homology to cover precisely this gap, and trace the phylogeny of the female orgasm. As we will see in the next section, the incorporation of new homology concepts has been instrumental in this new move permitting to connect the physiology of human pleasure with that of other species.

## 7 BACK TO PHYLOGENETIC HOMOLOGY: THE OVULATORY‐HOMOLOG HYPOTHESIS

Evolutionary explanations have tended to implicitly or explicitly consider the female orgasm as a uniquely human (or, at most, primate) trait. In a series of recent papers, Mihaela Pavličev and Günter Wagner have revised this assumption and revisited the evolutionary enigma of the female orgasm from a comparative scope (Pavličev & Wagner, 2016; Wagner & Pavličev, 2017; Pavličev et al., 2019). Pavličev and Wagner follow Lloyd's critique of adaptationism and bet for “homology thinking” as an alternative explanatory approach to the evolution of the female orgasm (Wagner & Pavličev, 2017, pp. 1–3). However, differently to Lloyd, they argue that extant theories (including both adaptive and byproduct theories) have been so far focused on the human orgasm, and remained incapable of tracing back the evolutionary origin of the trait. Instead, the elucidation of the origination of the female orgasm requires a *phylogenetic* notion of homology based on a comparison of this character across lineages. Under this premise, Pavličev and Wagner advocate for an alternative explanation of why the female orgasm has no evident reproductive functions in humans. According to their “ovulatory‐homolog” hypothesis, the female orgasm and the ovulation process were physiologically linked in an ancestral stage of mammalian phylogeny, but became later decoupled in eutherians.

In contrast to the well‐established anatomical and developmental data supporting sexual homologies, the postulation of a phylogenetic homology for the mammalian female orgasm required the collection of new experimental and phylogenetic evidence. In a recent experiment, Pavličev and collaborators showed that copulation‐induced ovulation in rabbits was affected by the administration of fluoxetine, a well‐known drug (the famous “Prozac) that inhibits orgasm in humans (Pavličev et al., 2019). Although these results do not rule out other explanations, such as a convergent mechanism being responsible for the response to fluoxetine, they provide strong support for the hypothesis that the female primate orgasm derives and still shares some mechanistic basis with the neuroendocrine reflex inducing ovulation in other mammals. Moreover, this experimental approach to homology represents a substantial step in testing homology‐based explanations of the female orgasm. From a phylogenetic perspective, the ovulatory‐homolog hypothesis is based on phylogenetic data suggesting that male‐induced ovulation is the ancestral condition in mammals, while spontaneous ovulation is a derived mode of the ovarian cycle originating later in several eutherian clades. More recently, Pavličev and collaborators have strengthened the phylogenetic support of the ovulatory‐homolog hypothesis with a comparative anatomy of the development of male and female external genitalia in different mammalian species (Pavličev et al., 2022). While after early joint development, male genital and urinary tracts always integrate into the phallus, in females there is a lot of structural and positional interspecific variation. In species with spontaneous ovulation, the clitoral glans tends to be far apart from the vagina and is not functionally linked to ovulation, while in species with copulation‐induced ovulation, the clitoral glans is generally located inside the vagina.

The individuation of the female orgasm as a comparable, mammalian trait, entails establishing homological relationships at different levels. Concerning morphological homology, the anatomy of the female orgasm has a major role in the postulation of the ovulatory homolog hypothesis. The comparative anatomy of female genitalia is an understudied topic that was hardly ever mentioned in connection to the evolution of the female orgasm until Pavličev and Wagner's research. The intermittent neglect of the clitoris has been widely documented in the history of human anatomy, and is still patent in recurrent omissions of the organ from contemporary anatomical drawings (Moore, 2018; Tuana, 2004). Recent studies describing the external and internal anatomy of the clitoris, including not only the clitoral glans, but also the paired bulbs and corpora, characterize their results as rediscoveries of forgotten anatomical works (O'Connell et al., 2005). This new research has raised interest among medical humanities and social science scholars, who have reflected on the consequences of considering the holistic nature of female pleasure for the debate on the locus of female orgasm (Blechner, 2017; Moore, 2018; Tuana, 2004). In the evolutionary terrain, a parallel debate on the role of clitoral anatomy has been raised in the reactions triggered by the ovulatory homolog hypothesis. Komisaruk (2016) pointed out that Pavličev and Wagner (2016) had not been careful enough in distinguishing between the external glans and the internal corpus of the clitoris, which can also be stimulated in copulation through the vaginal wall. In their reply, Wagner and Pavličev (2016) acknowledge that more anatomical knowledge of the evolution of clitoral anatomy is needed, but argue that the externalization of the clitoral glans from the vagina, as associated to the evolution of spontaneous ovulation, gives a robust anatomical support for their hypothesis.

Although a good deal of the debate on the female orgasm has concerned the anatomical locus of sexual stimulation, orgasm itself is not a morphological character. Rather, postulating the homology of the female orgasm among mammals involves identifying sameness at a physiological level. This entails a major difficulty in applying the homology concept, which has classically been used for morphological characters, to the female orgasm. Nonetheless, in the last years, various voices have argued that there is no need to restrict the concepts of homology and novelty to structural components. Activities (Love, 2007) or bodily functions (Brigandt, 2017) can be equally homologized. In defining orgasm as a neuroendocrine reflex triggered by clitoral stimulation, the homology of the mammalian orgasm illustrates this notion of complex bodily parts as composed of both structures and functions open to evolutionary modification (Brigandt, 2017). In this regard, the ovulatory homolog hypothesis moves the definition of the human female orgasm from a subjective psychophysiological experience to that of an objective, comparable trait. As mentioned above, the founders of modern sexology provided the first definitions of the human orgasm in terms of physiological homology. But in focusing on humans, they framed orgasm as an integrated step of a whole sexual response continuum, and therefore did not exclude the subjective dimension of this process. The individuation of the female orgasm as a neuroendocrine reflex makes it possible to abstract away the subjective dimension of the female orgasm, insofar as a subunit of the same process can be identified as homologous. This partitioning of the sexual response reflects the factorial or combinatorial nature of homology, and, therefore, the fact that homological relationships can be partial (Fusco, 2022).

The definition of female orgasm as a neuroendocrine reflex has been one of the most controversial issues in the scientific reception of the ovulatory homolog hypothesis, as shown in a recent article in *Scientific American* covering the reactions of two neuroendocrinologists to the experimental results on rabbits (Lewis, 2019). Julie Bakker, from the University of Liège, pointed out the limitations of studying orgasm in animal models: “There's no such thing as orgasm in rabbits”; “it is more like a light switch, in which male stimulation triggers the brain, which triggers ovulation”. In the same line, Raúl Paredes, from the National Autonomous University of Mexico, blames as reductionistic the definition of orgasm as an induced reflex: “This is a human construct because, aside from the physiological changes that can occur during sex, the definition involves feelings of pleasure” that “can't be measured in animals”. In a response paper, Wagner and Pavličev (2017) argue that, in relying on subjective experiences and peripheral signs of excitement, purported definitions are rather descriptions, of the female orgasm. Defining orgasm as a neuroendocrine reflex they argue is the only way to provide an objective, comparable definition allowing us to trace back its evolutionary origin.

The search for the evolutionary delimitation of the female orgasm not only shows how definitions of a character change substantially under different notions of homology. It illustrates as well the interdependency between the homology and the novelty concepts. Is the female primate orgasm an evolutionary novelty with no evolutionary precedent in the mammalian lineage, or rather a character state derived from the ovulatory reflex in ancestral species with coitus‐induced ovulation? (Wagner & Pavličev, 2017). Depending on whether the human female orgasm is individuated as a novelty or as a derived character, its phylogenetic origin might be traced back to primates, to mammals with spontaneous ovulation, to the ancestor of mammals, or even to the origin of amniotes.

Following the reasoning that the female orgasm was ancestrally connected to ovulation, a recent paper suggests that this trait could indeed be traced back to the reflex of ovulation in the transition from external to internal fertilization (Lodé, 2020). Most evolutionary biologists would likely identify the phylogenetic origin of the male orgasm in the origination of amniotes, when the developmental structure for both the penis and the clitoris originally evolved, together with internal fertilization (Sanger et al., 2015). Hence, the assumption that the female orgasm originated in mammals, the male orgasm being much more ancestral, might be revised. If the reflexes of ovulation and ejaculation are proved to share evolutionary roots, one might end by harmonizing phylogenetic and sexual homology in a unitary explanation for the evolution of orgasm. Less ambitiously, the ovulatory homolog hypothesis assumes that orgasm is at least present in mammals. Therefore, the female primate orgasm seems to be understood as a character state of a single, homologous trait that has undergone evolutionary modifications, including a disentanglement between female orgasm and ovulation in eutherians. After this evolutionary autonomisation from ovulation, the female orgasm would qualify as an evolutionary innovation. Nonetheless, the externalization of the clitoral glans and the evolution of spontaneous ovulation have independently evolved multiple times in mammals (Pavličev & Wagner, 2016). Accounting for the origination of these homoplastic traits might therefore require additional explanations. In particular, one might argue that the specificity of female sexuality in primates is not only determined by the decoupling between orgasm and reproduction, but rather by psychosocial factors. In primates, female sexuality is more influenced by cognitive and relational aspects, and extended female sexuality, as connected to the evolution of hidden ovulation, reaches a unique scope in humans (Thornhill & Gangestad, 2008).

It is generally assumed that selection‐based and homology‐based explanations constitute different explanatory agendas addressed to solve nonoverlapping problems in evolutionary biology (Amundson, 2005). Just like adaptive hypotheses do not aim at accounting for the origination of the female orgasm, the individuation of the female orgasm as the neuroendocrine mechanism decoupled from induced ovulation does not provide the adaptive causes for its evolutionary persistence. Instead, the aim of homology‐based explanations is to shed light on the origination of the female orgasm and individuate this trait as a historical unit. Indeed, an explicit conceptual distinction motivating the ovulatory homolog hypothesis is that between evolutionary explanations of the origin of a trait, and those accounting for the maintenance of this trait. From this perspective, extant hypotheses on the evolution of the female orgasm might not be incompatible, but rather refer to different stages of the evolutionary history of the trait. Thus, the decoupling between orgasm and conception would account for the origination of the female orgasm, the byproduct hypothesis might provide the developmental mechanisms for the maintenance of correspondences between the anatomy and physiology of pleasure in the two sexes (see Davis, 2019), and adaptive explanations would unravel the selective forces behind its evolutionary persistence. Nonetheless, mechanistic approaches to character evolution can also improve adaptive explanations, and even open new terrains for adaptive hypotheses. For instance, several studies have documented a prevalence of sexual symptoms during menopause in humans, including poor arousal and orgasm (e.g., Nappi & Lachowsky, 2009). This association might be interpreted as supporting the hypothesis on the ancestral connection between orgasm and ovulation, but the tenacity of orgasm, namely the fact that it does not disapear with the cessation of ovulation despite a decrease in intensity, might also indicate that orgasm has other functions not related to reproduction, or support the non‐function claims of the byproduct hypothesis. At this point, it is important to emphasize that physiological and evolutionary functions are not synonymous. The evolutionary function of the uterus is obviously related to reproduction, but females do not lose their uterus once they reach menopause and lose their physiological ability to reproduce. Many traits are only functional during some stages of the life cycle, and are evolutionarily preserved for that reason. But what matters for our argument is that the postulation of an ancient evolutionary association between ovulation and female orgasm opens the way for testing new mechanistic hypotheses on, for instance, the physiological connection between menstruation and orgasm. Moreover, the hypothesis on the independent evolution of the female orgasm requires unraveling the evolutionary forces behind the evolution of spontaneous ovulation in mammals and the associated changes in genital anatomy.

## 8 CONCLUSIONS

The female orgasm is one of the most contested topics on human sexuality, charged with speculation, storytelling, and gender biases. At this crossroads, shifts in the reference of comparison and the application of different definitions of homology to the understanding of sexual anatomy and pleasure have been the underlying conceptual foundation for theories on female sexuality. Sexual homology was the prevalent model for understanding the physiology of female pleasure until the mid‐19th century, when the rupture of the link between sexual pleasure and reproduction became the main foundation for the distinctiveness of sexuality in women. At the turn of the new century, two different disciplines concerned with the study of sexuality, namely psychoanalysis and evolutionary biology, explored different strategies to relink sexuality and reproduction in the female body. This reinstated link reframed the contrast between males and females under a new model of sexual complementarity, where homological relationships were downgraded and the two sexes played the role of matching pieces in the puzzle of reproduction. The concept of sexual homology recovered its centrality in the mid‐20th century, becoming the cornerstone in defense of egalitarian sexuality in the new science of sexology, and later on in evolutionary biology under the byproduct hypothesis. Recent research on the evolutionary origin of the female orgasm challenges this continuous trend of portraying female sexuality as only deriving from sexual homology. In linking the human female orgasm to females of other species, instead of taking male anatomy and pleasure as the sole reference for comparison, the female orgasm is individuated as a relatively autonomous evolutionary domain not necessarily coupled to reproductive functions. Moreover, current competing explanations of the evolution of female orgasm, which appear to contradict one another, may actually be reconcilable because they refer to different stages of the evolutionary history of the trait.

Our survey on the evolutionary explanations of female pleasure shows that the perception of the female orgasm as a mysterious, or even a paradoxical trait, is biased by cultural expectations about female pleasure. The expected link between sexuality and reproduction in women has been a core driving force of this riddle, and evolutionary biologists have further promoted this narrative by competing for a resolution to their created puzzle. Nonetheless, social biases in biological studies of the female orgasm do not only derive from the assumption of a tight link between sexuality and reproduction in women. Social expectations on the egalitarian nature of human sexuality have also played a role in characterizing as paradoxical the lack of reproductive function of the female orgasm. In breaking the necessary link between female pleasure and reproduction, new biological research on female orgasm also challenges the perception of biology as a source of essentialist associations between female sexuality and reproduction. These two dimensions of female physiology might be historically linked, but evolution itself broke this connection in our more recent history. As Halperin outlined decades ago, the paradigm of ‘masculinity’ was defined by the ability of men to isolate sexual pleasure and reproduction, even when only in men recreative and procreative sex are physiologically linked (Halperin, 1990, p. 285). Despite the fact that evolutionary hypotheses themselves do not imply any value statement about female sexuality (Lloyd, 2005; Wagner & Pavličev, 2016), unraveling the evolutionary origins of the decoupling between sexual pleasure and reproduction shows how biological theories of human sexuality do not only constrain but also provide new anthropological imaginaries for theorizing femininity.

## AUTHOR CONTRIBUTIONS

**Silvia Basanta**: Conceptualization; writing – original draft. **Laura Nuño de la Rosa**: Conceptualization; writing – original draft.

## Data Availability

We only use bibliographic data.

## Footnotes

1 The history of gynecology is a good trace of these progressive efforts for understanding sexual difference. Although the first uses of the word “gynecology” show up in the 18th century (McGrath, 2002), the emergence of gynecology as a medical discipline specifically addressed to study the female body has a long history (King, 2017). The progressive birth of gynecology had important consequences for the representation of sexual difference. While male bodies were the subject of the research on universals (reason, speech or posture), the study of female bodies was focused on their sexual and reproductive traits (Schiebinger, 2004). Nonetheless, the meaning attributed to female genital and reproductive organs throughout the 18th and 19th centuries captured a much larger span of disorders and pathologies than what is today captured by the discipline of gynecology. Before the emergence of the modern surgical discipline after 1850, a very wide variety of physiological and cognitive traits in women were attributed to their genital organs, based on the idea that women's reproductive functions dominated their entire being.

2 As we will see later, contemporary discussions show that the distinctiveness between developmental and historical homology concepts, as applied to serial characters, is not so neat as Ghiselin expected (Fusco, 2022).

3 As an illustration, sexual promiscuity was regarded as the primitive state in human history, followed by a later control of sexuality, and sexual pathologies were read as atavisms (Moore, 2021).

4 This included some exceptional psychoanalysts (Sherfey, 1966), but the most influential essay was “The Myth of the Vaginal orgasm,” published in 1970 by Anne Koedt (1970) in a radical‐feminist journal. Citing Masters and Johnson, Koedt blamed Freud's “invention” of the vaginal orgasm and the devastating psychological consequences that diagnoses of frigidity had for women. In turn, Koedt's essay inaugurated a further historiographical myth in post‐1970s feminist vindications of the clitoral orgasm, according to which the clitoral orgasm had been broadly repressed in the late 19th‐early 20th century.

5 In the 1920s and 1930s, endocrinologists discussed orgasm in rabbits in connection to induced ovulation (Marshall & Verney, 1936; Walton & Hammond, 1928), and sperm transport (Parker, 1931).

6 It has been argued that the conflation between orgasm and conception has led to important misconceptions on the male side as well. Orgasm and ejaculation are also different physiological phenomena in males, and only seminal emission, not orgasm itself, is essential to conception. Therefore, “there is no better adaptational explanation for the existence of men's orgasm than for the existence of women's” (Komisaruk, 2006, p. 12).
